# A mixed-methods longitudinal study of Marshallese infant feeding beliefs and experiences in the United States: a study protocol

**DOI:** 10.1186/s13006-021-00412-1

**Published:** 2021-08-28

**Authors:** Britni L. Ayers, Cari A. Bogulski, Lauren Haggard-Duff, James P. Selig, Pearl A. McElfish

**Affiliations:** 1 College of Medicine, University of Arkansas for Medical Sciences Northwest, Fayetteville, AR USA; 2 Office of Community Health and Research, University of Arkansas for Medical Sciences Northwest, Fayetteville, AR USA; 3 College of Nursing, University of Arkansas for Medical Sciences Northwest, Fayetteville, AR USA; 4 Fay W. Boozman College of Public Health, University of Arkansas for Medical Sciences, Little Rock, AR USA

**Keywords:** Infant feeding, Marshallese, Pacific Islander, Community-based participatory research

## Abstract

**Background:**

Arkansas has the largest population of Marshallese Pacific Islanders residing in the continental United States. Marshallese are disproportionately burdened by poorer maternal and infant health outcomes. Exclusive breastfeeding can prevent or help mitigate maternal and infant health disparities. However, exclusive breastfeeding among United States Marshallese communities remains disproportionately low, and the reasons are not well documented. This paper describes the protocol of a mixed-methods concurrent triangulation longitudinal study designed to explore the beliefs and experiences that serve as barriers and/or facilitators to exclusive breastfeeding intention, initiation, and duration among Marshallese mothers in northwest Arkansas.

**Methods:**

The mixed-methods design collects qualitative and quantitative data during simultaneous data collection events, at third trimester, six weeks postpartum, and six months postpartum. Quantitative and qualitative data will be analyzed separately and then synthesized during the interpretation phase. The research team will disseminate results to study participants, research stakeholders, the broader Marshallese community, and fellow researchers.

**Discussion:**

Findings and results will be presented in subsequent manuscripts upon completion of the study. This study will be an important first step to better understand beliefs and experiences to exclusive breastfeeding intention, initiation, and duration in this community and will inform tools and interventions to help improve health outcomes. The study will also aid in filling the gap in research and providing essential information on the infant feeding beliefs and barriers among a Marshallese community in Arkansas.

**Supplementary Information:**

The online version contains supplementary material available at 10.1186/s13006-021-00412-1.

## Background

Marshallese are a Pacific Islander population experiencing significant health disparities both in the United States (US) and globally. Cardiometabolic diseases are particularly high in Pacific Islander populations [1–17]. The 2010 Centers for Disease Control and Prevention (CDC) survey showed that high percentages of Pacific Islander respondents in the US had experienced heart disease (20%), hypertension (41%), and stroke (16%), higher than any other racial/ethnic group [1]. Additionally, the CDC’s National Health Interview Survey documented that 44% of Pacific Islanders surveyed in 2010 were obese, compared to 37% of African Americans and 32% of Hispanic/Latin [1]. Nationally, only 19% of Pacific Islanders reported healthy body mass indexes (BMI). A study with adult Marshallese in Arkansas demonstrated that (*n =* 401) 41% had blood pressure measures indicating hypertension, 16% had prehypertension, and 90% were overweight or obese. Additionally, diabetes screenings indicated that 38% had HbA1c levels indicative of diabetes, and 33% had levels indicative of prediabetes [18]. Little is known about childhood obesity among Pacific Islander children. The available data shows Pacific Islander children from 12 to 17 have obesity prevalence ranging from 15 to 36% [19, 20].

Exclusive breastfeeding is recognized as the best source of nutrition for infants. Not exclusively breastfeeding increases the risk of obesity and cardiometabolic disease for both mother and infant [21–29]. Exclusive breastfeeding provides protective factors against obesity in children long-term (into adulthood) [30]. Studies show that higher plasma-insulin concentrations and higher protein intake, in infant formula-fed compared to breastfed infants, stimulate fat deposition, signifying that exclusive breastfeeding has protective effects against obesity [30, 31]. In addition to significantly increasing the risk of childhood obesity, not exclusively breastfeeding increases an infant’s risk of sudden infant death syndrome, as well as acute gastrointestinal and respiratory infections [21–23, 29]. For the mother, not exclusively breastfeeding can increase the risk of metabolic syndrome and type 2 diabetes [24–28] which can increase obesity in subsequent children [30].

Exclusive breastfeeding rates among Pacific Islanders in the US are lower than in other populations [32]. Most concerning, the limited research available shows a decline in exclusive breastfeeding rates after women migrate to the US. Only 12% of Pacific Islander infants in the US are exclusively breastfed at six months compared to 31% of infants in the Marshall Islands and 25% for other populations in the US [33].

It is not clear what factors affect exclusive breastfeeding in Pacific Islander subgroups in the US. This article describes the protocol of a mixed-methods longitudinal study designed to understand beliefs, experiences, and perceptions that serve as barriers and/or facilitators of exclusive breastfeeding intention, initiation, and duration in Marshallese women. This study is an important first step toward developing culturally relevant, tailored interventions to improve the prevalence of exclusive breastfeeding among the Marshallese and other Pacific Islanders in the US.

## Methods

### Study aims

The aims of this study are to explore the beliefs, perceptions, and experiences that serve as barriers and/or facilitators to exclusive breastfeeding intention, initiation, and duration among Marshallese mothers in northwest Arkansas at multiple social-ecological levels using longitudinal, in-depth qualitative interviews.

### Approach

A community-based participatory research (CBPR) approach will be used in the design and implementation of this study. CBPR is an approach to honor and integrate Marshallese cultural values and practices into every aspect of the research [34–41]. A CBPR approach allows community knowledge to inform all aspects of the research to be more culturally-acceptable and has demonstrated effectiveness in building alliances to improve health when disparities result from systematic disadvantage, racism, and historical trauma [34–41]. To ensure cultural appropriateness, this study is guided by the Healthy Start Community Action Network (CAN) that includes local healthcare professionals, Marshallese community members, and an interprofessional research team. The research team includes quantitative and qualitative researchers as well as Marshallese bilingual study staff to provide feedback of study materials and input on how to modify the study materials and protocol to be culturally-appropriate for Marshallese participants [42].

A mixed methods design will be utilized as an exploratory method to better understand beliefs and experiences to exclusive breastfeeding intention, initiation, and duration of the Marshallese living in northwest Arkansas. This design collects qualitative and quantitative data during simultaneous data collection events at third trimester and six weeks postpartum. The six months postpartum data collection event is a qualitative interview only. Quantitative survey data will not be collected at six months postpartum and will instead occur as a follow up phone call to assess if participants are continuing to breastfeed.

### Data collection

Data collection began in October 2020. Data (see instruments below) will be collected at third trimester, six weeks postpartum, and six months postpartum. These data collection time points were chosen to identify beliefs and experiences with exclusive breastfeeding intention, initiation, and duration over time [32, 43]. Participants will also be provided the option to sign a Health Insurance Portability and Accountability Act (HIPAA) release to access their maternal and neonatal medical records which will be obtained to abstract clinical information after the participant gives birth at six weeks postpartum. The research staff will carefully monitor study procedures to protect the safety of research subjects, the quality of the data, and the integrity of the study. No identifiable data will be collected. All data collected will be kept in password-protected files, organized in dated folders, and stored in locked cabinets at the University of Arkansas for Medical Sciences (UAMS) Northwest office. In case of electronic files, these may be kept in secure UAMS servers and/or databases.

### Instruments

The quantitative surveys and qualitative interview guides were developed with intensive input from Marshallese stakeholders and are specific to this study. After the instruments were initially drafted with stakeholders, the CBPR team met monthly with three female Marshallese bilingual study staff who will be implementing the data collection in-language with the participants.

#### Quantitative surveys

The surveys will be implemented using Research Electronic Data Capture (REDCap) [44]. Each survey will take approximately 15–30 min to complete. There are two surveys: 1) a demographic survey given at the first data collection at third trimester; and 2) a Birth Satisfaction Survey (BSS) given at six weeks postpartum (Table 1). The BSS is a self-report questionnaire, targeting satisfaction with labor and birth [45]. The original 30-item scale has been revised to ten questions (Birth Satisfaction Survey-Revised [BSS-R]) and has been recommended as the instrument of choice for global use to assess maternal birth experience.

**Table 1:** Data collection timeline

Timeline	Third trimester	Six weeks postpartum	Six months postpartum
Consent and enrollment	X		
Demographic survey	X		
Birth Satisfaction Survey		X	
Individual interview	X	X	X
Birth record abstraction		X	

Community stakeholders and bilingual Marshallese study staff reviewed the surveys, and both instruments went through three iterations [45].

#### Qualitative interview guides

Qualitative interviews will take approximately 30 min to an hour and will occur at all three data collection events (Table 1). Interview guides have six domains: 1) infant feeding intent; 2) birth experience; 3) feeding infant; 4) family’s role in feeding baby; 5) barriers and facilitators to infant feeding; 6) public experiences to infant feeding; and 7) breastfeeding duration (see Prenatal and Postpartum Interview Guide, Additional file 1). The interview guide went through five iterations of revisions with the Marshallese stakeholders to ensure cultural relevance.

#### Maternal and neonatal medical records

The research team will abstract medical record information about the mother and infant. The data abstracted from the mother will include: 1) date of first prenatal care; 2) number of prenatal care visits; 3) fasting glucose; 4) blood pressure; 5) gestational weeks at delivery; 6) complications; 7) gestational diabetes mellitus test results; 8) mothers’ pre-maternal weight status; 9) the amount of weight gained (both cumulatively and per trimester); 10) timing of weight gained; and 11) infant feeding initiation. For infants, data abstraction will include: 1) weight and height of infant; and 2) birth/medical complications.

After qualitative and quantitative data collection instruments were confirmed by community stakeholders, Marshallese bilingual study staff translated the instruments into Marshallese. After translations were complete, the CBPR team met with the Marshallese bilingual study staff and conducted mock data collection events biweekly over the course of four months. These mock data collection events served as training for the Marshallese bilingual study staff and allowed the team to evaluate any challenges in cultural nuance, comprehension, and translations. When conducting CBPR, it is critical to allow for time and flexibility to guarantee the study and data instruments are culturally appropriate to the target community [46].

### Recruitment, consent, and retention

All study information documents used for recruitment, consent, and retention were developed in collaboration with Marshallese stakeholders (CAN and Marshallese bilingual study staff) using a CBPR approach [47]. Thirty women will be recruited by the Marshallese bilingual study staff, who will recruit at local clinics, faith-based organizations, and community-based organizations.

The inclusion criteria are: 1) women who self-report as Marshallese; 2) 18 years of age or older; and 3) 28–39 weeks pregnant. Exclusion criteria are: 1) conception with the use of fertility treatments; 2) multiple gestations; and 3) use of medications known to influence fetal growth (e.g., glucocorticoids, insulin, thyroid, hormones). The target of thirty was chosen because it will allow us to reach saturation [48, 49]. Our previous work with Marshallese female participants has used 30 as a target enrollment number, and this has demonstrated effective in reaching saturation [48, 50, 51]. Further, our team works closely with a biostatistician who has verified this is an appropriate target enrollment number for exploratory analyses. If saturation is not achieved among thirty women, the CBPR team will recruit additional participants.

Potential participants who meet the inclusion criteria will be offered the opportunity to join the study and complete the consent process prior to completing the first data collection event. Trained bilingual female Marshallese study staff will conduct the consent process. The bilingual study staff will read the consent aloud to the participants in the participant’s language of choice (English or Marshallese). Participants will have the opportunity to have their questions answered prior to consent. Each participant will be provided a copy of the consent in either/both English and Marshallese.

The CBPR team will use an engaged approach to collaboratively develop a retention plan with Marshallese stakeholders. The retention plan specifies that all study staff responsible for retention will be bilingual (Marshallese/English). Marshallese bilingual study staff will obtain each participant’s contact information and preferred method of contact. Marshallese bilingual study staff will also collect contact information for at least two relatives and ask participants for permission to contact their relatives if needed. Confidentiality rules will be followed, and no participant information will be provided to relatives. Before each data collection visit, bilingual study staff will contact study participants about the upcoming data collection visit. If a participant withdraws, the study team will document who withdrew and why they withdrew. Participants will receive a $40 Walmart gift card at each data collection event.

### Setting

To remove the barrier of transportation, the data collection will occur at the Jones Center in Springdale, which has the highest Marshallese population in northwest Arkansas. If transportation is still a barrier for participants, bilingual study staff will pick up participants prior to the data collection and transport to the Jones Center. Participants may bring their infants and/or children to the data collection event if needed, and additional study staff will provide infant and/or childcare.

### Data analysis

#### Quantitative surveys

Quantitative data analysis of the survey results will utilize descriptive techniques. The descriptive analyses will utilize frequencies and proportions to summarize the exclusive breastfeeding intentions, initiation, and duration of Marshallese mothers living in northwest Arkansas, which have not been systematically explored prior to this study. Analysis will report the total number of participants included and will include all non-missing valid responses from all participants, regardless of the presence of missing data on other survey items. For analyses examining changes in participants’ exclusive breastfeeding intentions, initiation, and duration over time, only those participants who participated in all relevant data collection events will be included.

#### Qualitative interviews

Qualitative data from interviews will be audio recorded and transcribed verbatim in the language it was spoken by participants. A bilingual Marshallese study staff will complete the transcription. Transcripts that are in Marshallese will be translated into English. Three trained researchers in qualitative analyses will start with initial coding, which consists of naming each data segment with short summations. This process helps organize the data for focused codes. The focused codes that emerge will be used to identify and develop the most salient categories within the data [52, 53]. The research team will discuss the emergent themes to ensure scientific rigor and inter-coder agreement. There will be two primary coders and one confirmation coder. The qualitative analytic approach will integrate inductive and deductive techniques. A codebook will be developed, and member checks with Marshallese bilingual study staff who collected the data will be implemented. Member checking with Marshallese bilingual research staff is a validation technique to explore the credibility of results and is critical in CBPR qualitative research [54]. Research staff trained in qualitative research will collaboratively discuss the themes to ensure scientific rigor and inter-coder agreement. The coding will aid in linking concepts and theme [55, 56]. Detailed record-keeping will provide an audit trail to increase confirmability.

In knowing the focus of the research, participants may temper their responses to make them more socially acceptable. To mitigate measurement bias, research staff will frame questions to be open-ended to prevent participants from simply agreeing or disagreeing and to guide the participant to provide a truthful and honest answer.

### Dissemination plan

Effective dissemination is crucial to achieving research impact and is a key component to conducting CBPR. The research team will disseminate aggregated results to study participants, research stakeholders (clinics, faith-based organizations, and community-based organization), the broader Marshallese community, and fellow researchers. Results will be disseminated to study participants and researcher stakeholders through a one-page summary that shows the aggregated research results using plain language and infographics. The summary will be posted on our website and Facebook pages. In addition, a town hall meeting will be held which invites the broader Marshallese community, clinics, faith-based organizations, and community-based organizations. No individual participant information will be shared, and all confidentiality procedures will be maintained. The data will be published in peer-reviewed journal articles and presented at academic conferences.

## Discussion

Marshallese are disproportionately burdened by cardio-metabolic outcomes. Exclusive breastfeeding can prevent or help mitigate these disparities. However, exclusive breastfeeding among US Marshallese communities remains disproportionately low and has not been well understood. To attend to the challenge of data collection during the COVID-19 pandemic, research protocols have been updated to include remote data collection via the phone if needed. This study will be an important first step to better understand beliefs and experiences to exclusive breastfeeding intention, initiation, and duration in this community and will inform tools and interventions to help improve health outcomes.

## Supplementary Information

**Additional file 1.** Prenatal and Postpartum Interview Guide.

## Data Availability

Not applicable.

## Abbreviations

BMI: Body Mass Index
BSS-R: Birth Satisfaction Survey-Revised
BSS: Birth Satisfaction Survey
CAN: Community Action Network
CBPR: Community-Based Participatory Research
CDC: Centers for Disease Control and Prevention
HIPPA: Health Insurance Portability and Accountability Act
REDCap: Research Electronic Data Capture
UAMS: University of Arkansas for Medical Sciences
US: United States
