# Supplementary material for: A mixed-methods longitudinal study of Marshallese infant feeding beliefs and experiences in the United States: a study protocol
Source: Int Breastfeed J. 2021 Aug 28;16:64. doi: 10.1186/s13006-021-00412-1 (PMC8401343; doi:10.1186/s13006-021-00412-1)
Supplement: Supplementary file 1 — Additional file 1. Prenatal and Postpartum Interview Guide. [file 13006_2021_412_MOESM1_ESM.docx]

**Prenatal and Postpartum Interview Guide**

| **Prenatal Interview Guide**  *Infant Feeding Intent*   1. How do you plan to feed your infant? Ewi wāwein am lemnak in naaj najidrik nin̄nin̄ eo nejūm?    1. Probe: Exclusive breastfeeding? Duration? Kaninnin wōt? Ewi aetokan?    2. What do you think of women who exclusively breast feed their babies? Ta am lemnak kin kōra ro rej kaninnin wōt niñniñ ko nejier im jab kōbato?    3. What do you think of women who do not exclusively breast feed their babies? Ta am lemnak kin kōra ro rejjab kaninnin niñniñ ko nejier ak rej kōbato? 2. You said earlier that you plan to feed your baby X. What contributed to your decision on how to feed your baby? Kwar ba moktalok ke kwōj lemnak in naaj najidrik rot io X niñniñ nejūm. Etke kwar lemnak in najidrik rot in? 3. What does your family think about exclusive breast feeding? TA an baamle eo am lemnak ikijjien kaninnin wot? 4. What does the Marshallese community think in general about exclusive breast feeding? Ta an jukjukinbed eo an Ri-Majōl lemnak ikijien kaninninwot?    1. How about Marshallese women in general? Ako baj kōra in Majōl ro?       1. How long do Marshallese women exclusively breast feed their infants? Ewi aetokan an kōra in Majōl kujon̄ kaninnin nin̄nin̄ ko nejier? 5. How do others influence how you feed your baby? Ewi wāwein an ro jet jelot wāwein am najidik niñniñ eo nejūm?   Probe: Church and church leaders Kabun̄ im ritel ro ie  Probe: Health care provider rijerbal ro ilo jikin taktō ko  Probe: WIC provider WIC  Probe: Workplace employer ro jen jikin jerbal  **Post-Partum Interviews: (8-20 weeks postpartum)**  *Birth Satisfaction*   1. Can you tell me about your birth experience? Komaron̄ ke kwal̗ok wāwein ko emoj am ioini ilo kemour?   *Feeding Your Baby*   1. How did you plan to feed your infant before he/she was born? How are you feeding your infant now? Ialmen am kar lomnak in niajdik ajiri eo nejūm mokta jen an kar lotak? Ialmen am najidik ajiri eo kiō?    1. What made you decide to feed your baby this way? Ta eo ear kōmman bwe kwōn najidik rōt in?       1. Hospital? Ewor ke jiban jen Hospital ikijjien wawein in?       2. Health care provider/Doctor? Rijerbal ro jen jikin taktō/Taktō eo rekar ke jiban?       3. WIC? WIC?       4. Marshallese community? Jukjuk in bed eo an RiMajōl?          1. Probe: Church and church leaders? Kabun kab ritel ro ie?       5. Workplace/employer? Jikin jerbal/ro kwoj jerbal nan er? 2. Where did you get your information to help you make that decision? Kwar ebbōk jen ia melele ñan jibañ kōmmane jokelet in am?   *Family’s Role in Feeding*   1. Who feeds the baby? Wōn ej najidik niñniñ eo? 2. Who takes care of the baby most of the time? Wōn eo ekka an lale niñniñ eo? 3. Who buys food for the baby? Wōn ej wia kijen niñniñ eo? 4. Who decides what the baby is fed? Wōn ej kelet ta ko niñniñ eo ej mōñā? 5. Whose opinions about feeding the baby are most important to you? Wōn eo elap am ronjake melele ko an ikijjien wawein najidrik niñniñ eo?   *Barriers and Facilitators*   1. Have you had challenges feeding your baby? If so, what? Enan̄in ke wor aban̄ ilo am najidrik nin̄nin̄ eo nejūm? Elan̄e aet, ta?   *Public Experiences*   1. What do you think about breast feeding in public? Ta am lōmnak ikijjien kaninnin ilobwilej?   Probe: Their own personal experience in public breastfeeding, comfort level in doing this. eñjake ko a make ikijjien kaninnin lobwilej, emmon ke enana? Ta ko kwoj enjaki ilo am kannin lobwilej? Emmōn ke, enana/ kwoj eliklik ke kwojjab?  *Feeding and Infant Health*   1. Can you describe some of the things that you think of when you think of a healthy baby? Komaron̄ ke kwalo̗k jet ian lemnak ko am ikijjien juōn niñniñ eo ejmour?    1. How do they act? Ialmen aer makūtkūt?    2. What do they do? Ta ko rej kōmmani? 2. How do babies tell you when they are hungry? Ialmen an nin̄nin̄ ko kwalo̗k ke rekwole? 3. How do you know when to feed the baby? Ialmen am jela ñaat eo kwōj aikuj najidik niñniñ eo? 4. How do you know when they are full? Ialmenam jela ke remat? 5. Where did you learn about how to feed your baby? kwar katak ia kilen najidik niñniñ eo nejūm?   *General Question:* Is there anything else about feeding babies in the Marshall Islands you would like us to know? Ewor ke bar jabdreiwōt ikijien wawein an ajiri mōn̄ā ilo Majōl im kwoj kōnan kwalok n̄an kim?  **Postpartum Interview (6 months)**   1. How are you feeding the baby now? Ialmen am najidik niñniñ eo nejūm kiō? 2. Can you tell me about your infant feeding experiences since the last time we talked? Komaron̄ ke kwalo̗k kilen am najidrik nin̄nin̄ eo nejum kio elikin arro kar kenaan eliktata? 3. Can you tell me about some challenges you have had trying to feed your baby? Komaron̄ ke kwalo̗k jet ian abañ ko emōj am ioni ilo am kajieoñ najidik niñniñ eo nejūm? 4. Who or what has been the most helpful in your infant feeding experiences? Wōn ak ta eo elap an jibañ ilo wawein am najidik ajiri eo nejūm? 5. Have you introduced solids to your infant? Konan̄in ke jino najidik ajiri eo nejūm mōñā ben?    1. If so, what have you introduced? Elañe aet, ta eo emōj am jino najdiki?    2. When did you introduce solids? Ñaat eo kwar jino najidik mōn̄ā ben?    3. What made you introduce those foods? Etke kwar jino najidiki mōñā kein? 6. Are there things you would change about your infant feeding experience? Ewor ke ta kwonaj kar ukōte ikijjien wawein am najidik ajiri eo nejūm?    1. If so, what and why? Elañe aet, ta im bwe? |
| --- |
